# Transcriptome profiling reveals the genetic basis of alkalinity tolerance in wheat

**DOI:** 10.1186/s12864-016-3421-8

**Published:** 2017-01-05

**Authors:** Chen Meng, Tai-Yong Quan, Zhong-Yi Li, Kang-Li Cui, Li Yan, Yu Liang, Jiu-Lan Dai, Guang-Min Xia, Shu-Wei Liu

**Affiliations:** 1 The Key Laboratory of Plant Cell Engineering and Germplasm Innovation, Ministry of Education, School of Life Sciences, Shandong University, Jinan, 250100 People’s Republic of China; 2 CSIRO Agriculture, GPO Box 1600, Canberra, ACT 2601 Australia; 3 Forest and Wetland Institute, Shandong Academy of Forestry, Jinan, 250014 People’s Republic of China; 4 Environment Research Institute, Shandong University, Jinan, 250100 People’s Republic of China

**Keywords:** Alkali tolerance, Digital gene expression, pH, Reactive oxygen species, Wheat

## Abstract

**Background:**

Soil alkalinity shows significant constraints to crop productivity; however, much less attention has been paid to analyze the effect of soil alkalinity on plant growth and development. Shanrong No. 4 (SR4) is an alkalinity tolerant bread wheat cultivar selected from an asymmetric somatic hybridization between the bread wheat cultivar Jinan 177 (JN177) and tall wheatgrass (*Thinopyrum ponticum*), which is a suitable material for studying alkalinity tolerant associate genes.

**Results:**

The growth of SR4 plant seedlings was less inhibited than that of JN177 when exposed to alkalinity stress conditions. The root cytosolic Na^+^/K^+^ ratio in alkalinity stressed SR4 was lower than in JN177, while alkalinity stressed SR4 contained higher level of nutrient elements than in JN177. SR4 plant seedlings accumulated less malondialdehyde (MDA) and reactive oxygen species (ROS), it also showed higher activity of ROS scavenging enzymes than JN177 under alkalinity stress. The root intracellular pH decreased in both alkalinity stressed JN177 and SR4, however, it was much lower in SR4 than in JN177 under alkalinity stress. The transcriptomes of SR4 and JN177 seedlings exposed to alkalinity stress were analyzed by digital gene expression tag profiling method. Alkalinity stress conditions up- and down-regulated a large number of genes in the seedling roots that play the functions in the categories of transcription regulation, signal transduction and protein modification.

**Conclusions:**

SR4 expresses a superior tolerance to alkaline stress conditions which is due to its strong absorbing ability for nutrient ions, a strong regulating ability for intracellular and rhizosphere pH and a more active ROS scavenging ability.

**Electronic supplementary material:**

The online version of this article (doi:10.1186/s12864-016-3421-8) contains supplementary material, which is available to authorized users.

## Background

Soil salinity and alkalinity both represent significant constraints to crop productivity. Nearly 830 million hectares of the land are affected by salt–alkali stress, and over 434 million hectares are alkalinized soils [1]. Alkaline salt stress and neutral salt stress are two distinct kinds of stresses for plants and should be called alkali stress and salt stress. Salt stress in the soil generally involves osmotic stress and ion injury, while alkali stress has an added high-pH effect. The high-pH directly affects the absorption of mineral elements and interferes with ionic balance. Thus, the effects of soil alkalinization on plants resulting from high concentrations of NaHCO_3_ and Na_2_CO_3_ may be more destructive than the effects of soil salinization induced by the accumulation of neutral salts, such as NaCl and Na_2_SO_4_ [2, 3]. The plant response to salinity stress has been exhaustively researched, but much less attention has been paid to analysing the effect of soil alkalinity on plant growth and development. The identification of alkali–responsive genes may provide valuable information on understanding the genetic basis of plants to alkali stress and improve alkali tolerance of crops by genetic engineering.

Wheat is an important crop in the world because of its high production (678 million tons in 2009, FAO), wide geographical range and high proportion of human consumption. Wheat is a glycophyte which is relatively sensitive to both salinity and alkalinity stress. However, two wheat lines, named as Shanrong No. 4 (SR4) and Shanrong No. 3 (SR3) were reported as salinity tolerant lines due to their more tolerant of salinity than JN177 [4, 5], which were selected from the derivatives of an asymmetric somatic hybridization between the salinity sensitive line, bread wheat cultivar Jinan 177 (JN177) and the highly tolerant related species, tall wheatgrass (*Thinopyrum ponticum*) [4, 5]. Transcriptomic and proteomic experiments showed that imposing salinity stress induced the differential up- or down-regulation of many genes present in SR3, and some of these, when expressed as transgenes, produced a marked positive impact on the plant’s level of salinity tolerance [6–11]. Although field trials showed that SR3 out-performs SR4 in pH neutral saline soils, the reverse was the case in alkaline pH saline soils (unpublished data). Thus, while SR3 had been registered as a salinity tolerant cultivar, SR4 was recommended for cultivation on high pH soils which is, therefore, a suitable material for identifying alkalinity tolerant associate genes.

Genome scale analysis of gene expression profiles is a powerful method to understand biological functions, such as cell differentiation, development and stress responses. With the increasing availability of sequencing data, expression profiling has been widely used in recent years to identify genes that are involved in the adaptive responses to drought and other abiotic stresses. High-throughput transcriptome sequencing and digital gene expression (DGE) tag profiling are efficient and cost-effective methods for characterizing non-model organisms without the need for a reference genome [12, 13]. DGE is particularly useful for identifying differentially expressed genes, thanks to its capacity to detect unknown and low abundance transcripts, and to generate absolute (rather than relative) transcript abundances [14].

Recently, some studies for alkaline-stress responses have been reported in *Glycine soja*, *Leymus chinensis* and *Tamarix hispida* [15–18]; however, there is still little information about the alkaline-stress responses of common wheat. The present study focuses on the short-term and more sustained responses of the wheat root to alkalinity stress, by contrasting the SR4 transcriptome with that of JN177 using DGE profiling method.

## Results

### The plant response to alkalinity stress

**Fig. 1:**
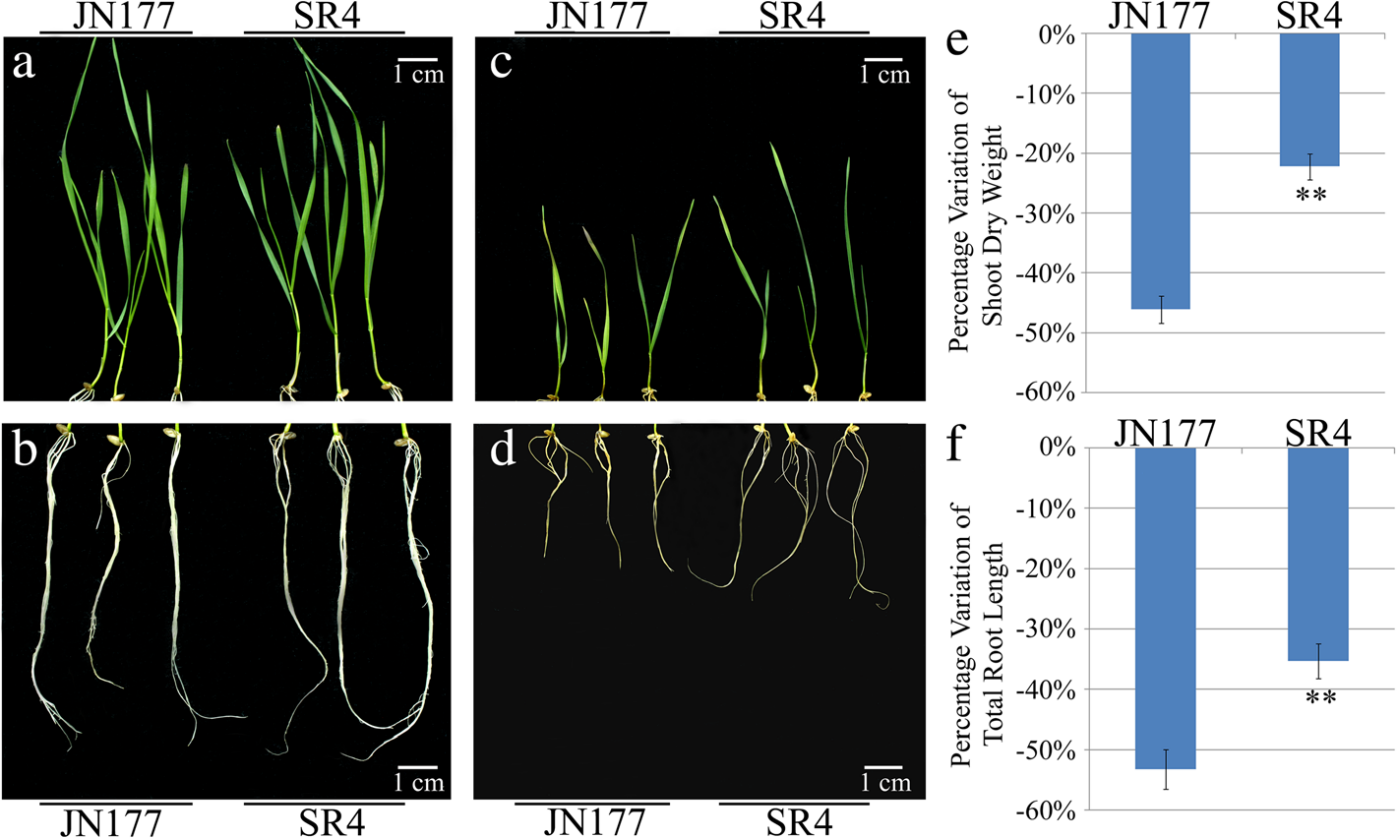
Growth of seedlings and roots of JN177 and SR4 under non-alkalinity and alkalinity stress conditions. **a**-**d** Three week old JN177 and SR4 seedlings grown under non-alkalinity stress (**a**, **b**) and under 100 mM alkali salts (**c**, **d**). Bar: 1 cm length. **e**-**f** The effect of alkalinity stress on shoot dry weight (**e**) and root length (**f**). Data are given in the form mean ± s.d. The double asterisks represent significant difference determined by the Student’s *t*-test at *P* < 0.01 (**)

The growth of seedlings and their roots of wheat line, SR4 was less inhibited than that of wheat cultivar JN177 when they were grown in the presence of the alkalinity stress. The growth of the seedlings and roots were similar between SR4 and JN177 when they were grown in the absent of the alkalinity stress (Fig. 1a and b). However, the shoot dry weight and root length were reduced to ~22 and ~35% respectively in SR4, while they were reduced to ~45 and 53% respectively in JN177 (Fig. 1c–f).

**Fig. 2:**
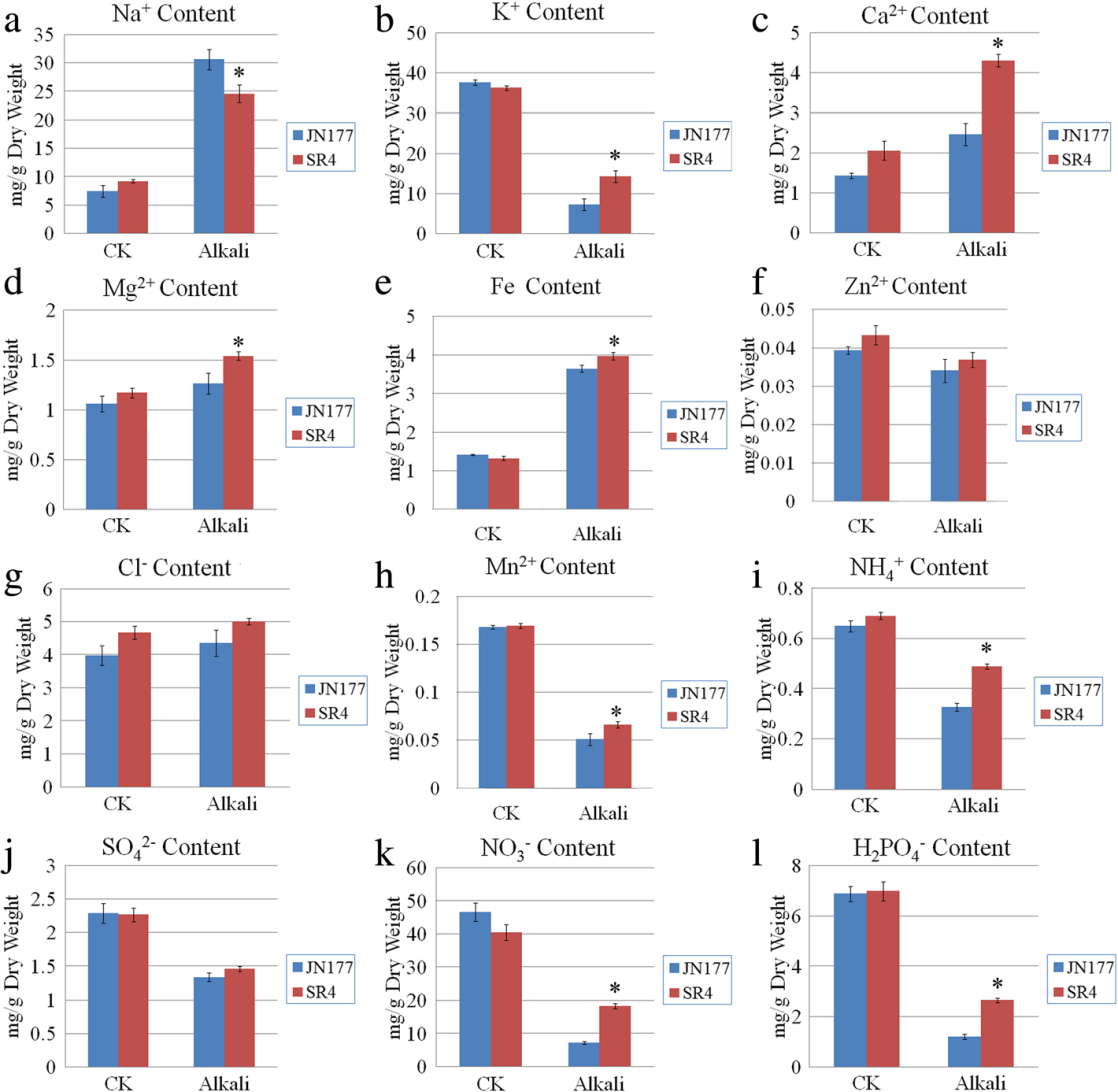
Content of selected ions of seedling roots of JN177 and SR4 under non-alkalinity and alkalinity stress conditions. Root content of (**a**) Na^+^, (**b**) K^+^, (**c**) Ca^2+^, (**d**) Mg^2+^, (**e**) Fe, (**f**) Zn^2+^, (**g**) Cl^−^, (**h**) Mn^2+^, (**i**) NH_4_^+^, (**j**) SO_4_^2−^, (**k**) NO_3_^−^ and (**l**) H_2_PO_4_^−^. Data are given in the form mean ± s.d. The asterisk represents significant difference determined by the Student’s *t*-test at *P* < 0.05 (*)

The high-salt environments in alkaline soil can break the ion homeostasis of plant cells; moreover, the high-pH caused by alkali stress directly affects the absorption of mineral elements. To understanding how SR4 adapt to overcome alkali stress, we analyzed the ion contents in roots of SR4 and JN177. The alkaline medium induced an increased Na^+^ and decreased K^+^ contents, overall lowering the K^+^⁄Na^+^ ratio in the roots of both wheats. The effect of the stress on root Na^+^ content was more marked for JN177 than for SR4, and vice versa for root K^+^ content (Fig. 2a and b). SR4 line maintained a higher root K^+^/Na^+^ ratio than that in JN177 cultivar. Root Ca^2+^, Mg^2+^ and total Fe concentrations were increased by the alkalinity stress in both cultivars, but SR4 maintained a higher level of root Ca^2+^, Mg^2+^ and total Fe content than that of JN177 (Fig. 2c–e). In contrast, alkali stress had little effect on the levels of Zn^2+^ and Cl^−^ in both cultivars (Fig. 2f and g). Alkali stress significantly decreased the levels of Mn^2+^, NH_4_^+^, SO_4_^2−^, NO_3_^−^ and H_2_PO_4_^−^ in roots of both cultivars, and the effect of the stress on root Mn^2+^, NH_4_^+^, NO_3_^−^ and H_2_PO_4_^−^ content was more marked for JN177 than for SR4 (Fig. 2h–l), indicating SR4 might absorb more nutrient elements than JN177 under alkali stress.

**Fig. 3:**
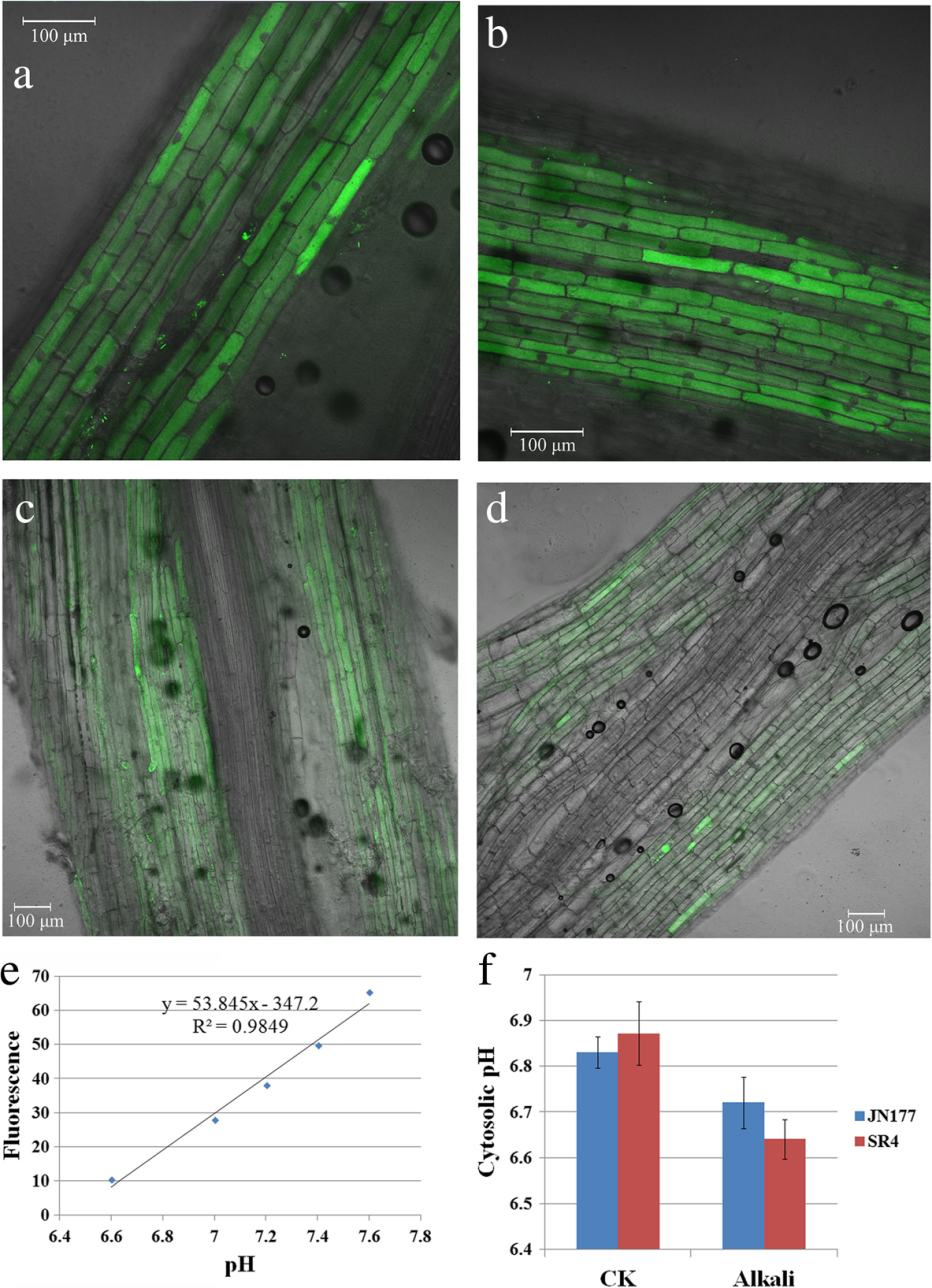
The intracellular pH of seedling roots of JN177 and SR4 under non-alkalinity and alkalinity stress conditions. **a**-**d** Fluorescence images for JN177 and SR4 under non-alkalinity stress (**a**, **b**) and under 100 mM alkali salts (**c**, **d**). **e** The fluorescence intensity is proportional to pH. **f** The intracellular pH of seedling roots of JN177 and SR4. Data are given in the form mean ± s.d

The high-pH caused by alkali stress can also directly affect the internal pH of the plant. To investigate how SR4 adapt to overcome the high-pH under alkali stress, we detected the intracellular pH in roots of both wheats by using pH indicator 2′7′-bis(2-carboxyethyl)-5(6)-carboxyfluorescein (BCECF). Under alkalinity stress, the BCECF fluorescence was decreased in root of both SR4 and JN177 compared to non-stressed condition, however, the fluorescence intensity was much lower in SR4 than in JN177 under alkalinity stress (Fig. 3a–d). Since the BCECF fluorescence intensity was proportional to the intracellular pH (Fig. 3e), we calculated the intracellular pH in root of both SR4 and JN177 according to BCECF fluorescence intensity. Results indicated that the intracellular pH fell about 0.1 and 0.2 pH unit in JN177 and SR4, respectively, under alkalinity stress (Fig. 3f).

**Fig. 4:**
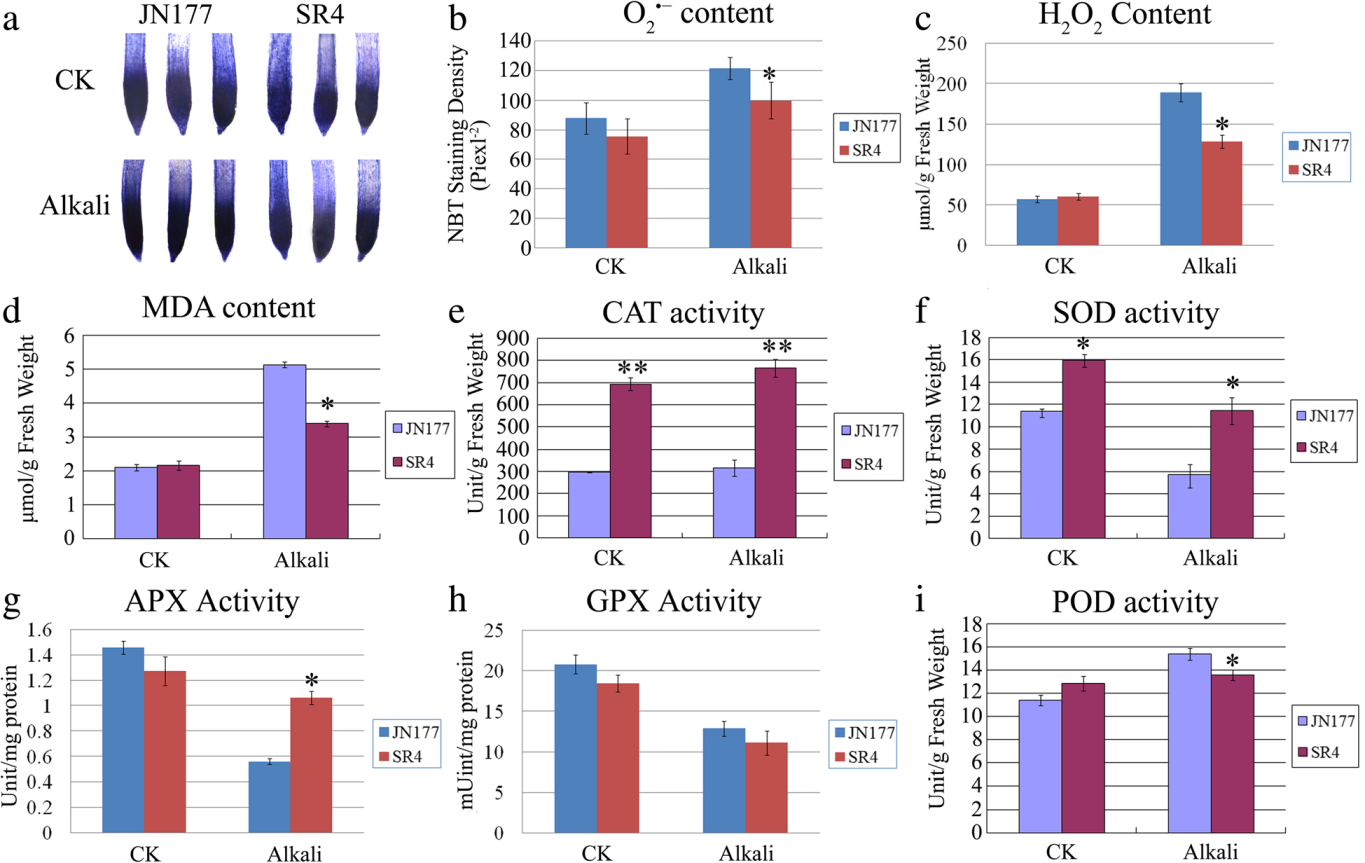
Content of ROS and activities of ROS scavenging enzymes of seedling roots of JN177 and SR4 under non-alkalinity and alkalinity stress conditions. **a** Root O_2_^•-^ content in three leaf stage seedlings grown in the absence (CK) or presence of 100 mM alkali salts. **b** The relative O_2_^•-^ content in the roots shown in (**a**) calculated from mean pixel density. **c**, **d** Root content of H_2_O_2_ (**c**) and MDA (**d**). **e**-**i** The activity of the ROS scavenging-associated enzymes in the seedling roots including catalase (CAT) (**e**), superoxide dismutase (SOD) (**f**), ascorbate peroxidise (APX) (**g**), glutathione peroxidise (GPX) (**h**) and peroxidase (POD) (**i**). Data are given in the form mean ± s.d. The asterisk and double asterisks represent significant difference determined by the Student’s *t*-test at *P* < 0.05 (*) and *P* < 0.01 (**), respectively

Environmental stresses including alkali stress can induce the production of toxic reactive oxygen species (ROS), which if not controlled, can pose a threat to cells. Thus, plants have developed antioxidant mechanisms to eliminate superfluous ROS under alkaline-saline stress, in which superoxide dismutase (SOD), ascorbate peroxidase (APX), glutathione peroxidase (GPX), peroxidase (POD) and catalase (CAT) are most important antioxidative enzymes. To assess whether SR4 can effectively scavenge ROS under alkali stress, we detected the ROS content and activity of major ROS scavenging enzymes in roots of both wheats. ROS can degrade polyunsaturated lipids to form malondialdehyde (MDA), which is generally taken as a marker of oxidative stress, thus we also detected the content of MDA in roots of both SR4 and JN177. Both O_2_^•−^ radicals and H_2_O_2_ was raised by alkali stress in wheats, and both cultivars accumulated similar level of ROS in the absence of alkalinity stress, but, in the presence of stress, SR4 accumulated ROS less than JN177 did (Fig. 4a–c). Similarly, root MDA content was raised by the stress, but less so in SR4 than in JN177 (Fig. 4d). With respect to the activities of the ROS scavenging enzymes SOD, APX, GPX, CAT and POD, the activity of CAT was consistently higher in SR4 than in JN177 (Fig. 4e); the activities of SOD, APX and GPX were inhibited by the stress, but the activity level of SOD and APX were consistently higher in SR4 than in JN177 (Fig. 4f–h); while the activity of POD was unaffected by the stress in SR4, but its activity was increased in JN177 (Fig. 4i). These results were fully consistent with the alkalinity tolerance profiles which showed that SR4 displaying a superior tolerance of alkalinity stress than JN177.

### Transcriptome profilings of wheat seedling roots under alkalinity stress

A total of 35,671,144 unfiltered tags were recovered from the six libraries (Table 1), with the number of sequences from each sample ranged from 5.7 to 6.2 million. After data filtering, a total of 33,994,682 clean tags were obtained for further analysis. The cleaned tags of each sample ranged from 5.4 to 5.9 million. About 50% of the clean tags can be mapped to the reference sequences from NCBI unigene databases, and most of them were uniquely mapped to gene tags (Table 1). All these unambiguous tags were mapped to over 20,000 unigenes (Table 1). The clean tags were also mapped to the wheat genomic DNA sequences from the IWGSC website (ftp://ftpmips.helmholtz-muenchen.de/plants/wheat/IWGSC/genePrediction_v2.2/). Although the percentage of the clean tags mapped to IWGSC wheat genome sequences (about 70%) were higher than that mapped to NCBI unigene databases (50%), the percentage of unambiguous tags mapped to the IWGSC wheat genome sequences (only 21–23%) were much less than that of NCBI unigene databases (42–46%) (Additional file 1: Tables S1). Since only unambiguous tags can be used for expression analysis, thus, the NCBI unigene databases were used for wheat RNA-seq tags annotation.

**Table 1:** Statistics analysis of the six DGE tag libraries constructed from the seedling roots of JN177 and SR4 mapping to NCBI unigene databases

Mapping to NCBI unigene databases	JNCK	JN0.5	JN24	SRCK	SR0.5	SR24
Raw data	6234376	5975104	5763389	5954683	5897848	5845744
Total number of clean tags	5943058	5712643	5487894	5692072	5633349	5525666
Clean tags mapped to gene	3184388	2806047	2666921	2837152	2745121	2771868
% of clean tags mapped to gene	53.58%	49.12%	48.60%	49.84%	48.73%	50.16%
Unambiguous tags mapped to gene	2741617	2430920	2317889	2460457	2379007	2388369
% of unambiguous tags mapped to gene	46.13%	42.55%	42.24%	43.23%	42.23%	43.22%
Unambiguous tag-mapped genes	21552	20185	20483	20663	20199	20648

*Note: JN* JN177, *SR* SR4, *CK* plants not subjected to alkalinity stress, 0.5 and 24: plants subjected to alkalinity stress for 0.5 h and 24 h, respectively

**Fig. 5:**
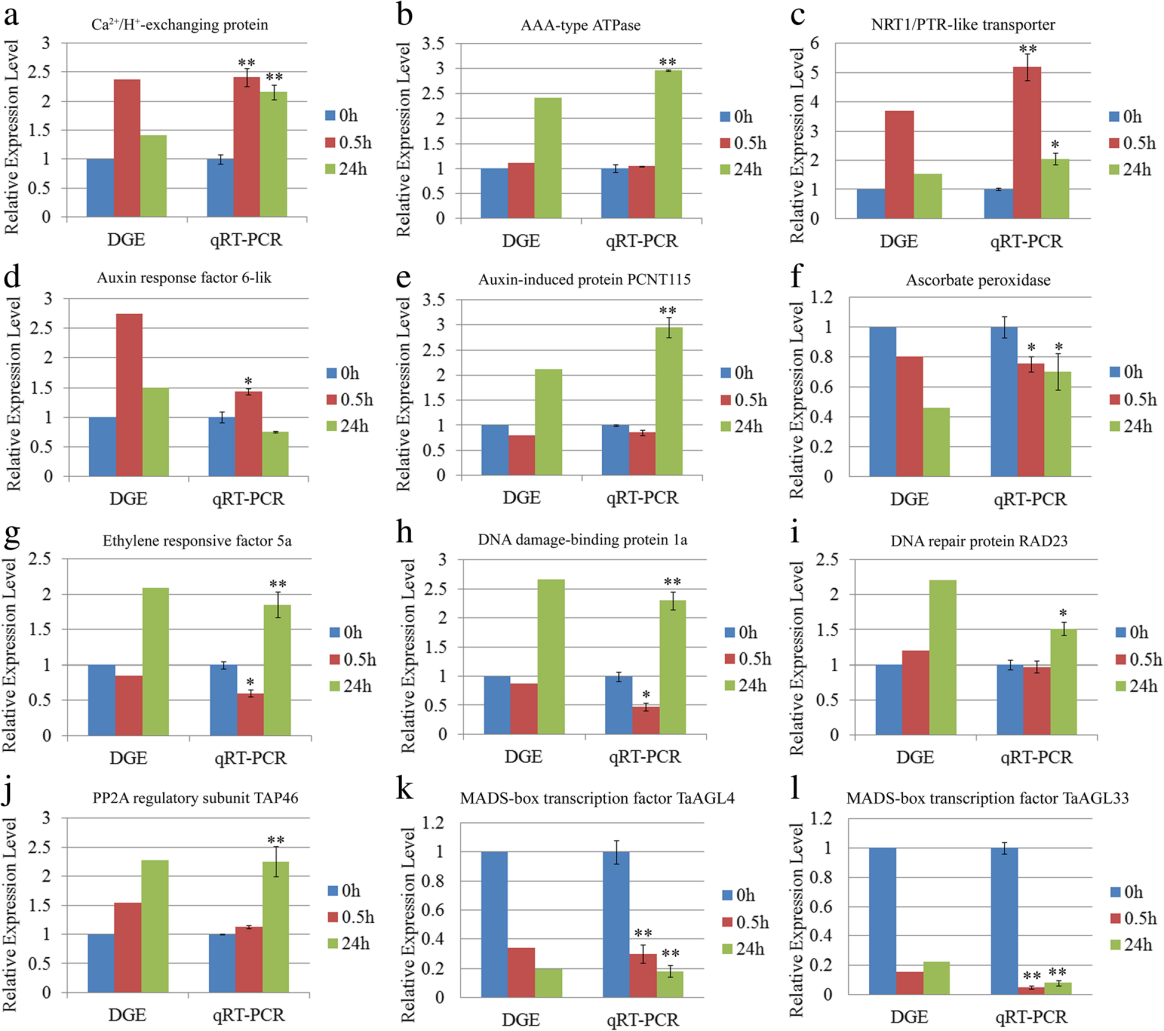
qRT-PCR-based validation of the transcription expression of twelve genes selected from the DGE analysis. **a** Ca^2+^/H^+^-exchanging protein. **b** AAA-type ATPase. **c** NRT1/PTR-like transporter. **d** Auxin response factor 6-like. **e** Auxin induced protein PCNT115. **f** Ascorbate peroxidase. **g** Ethylene responsive factor 5a. **h** DNA damage-binding protein 1a. **i** DNA repair protein RAD23. **j** PP2A regulatory subunit TAP46. **k** MADS-box transcription factor TaAGL4. **l** MADS-box transcription factor TaAGL33. The qRT-PCR data are given in the form mean ± s.d. (*n* = 3). The asterisk and double asterisks represent significant difference determined by the Student’s *t*-test at *P* < 0.05 (*) and *P* < 0.01 (**), respectively

**Fig. 6:**
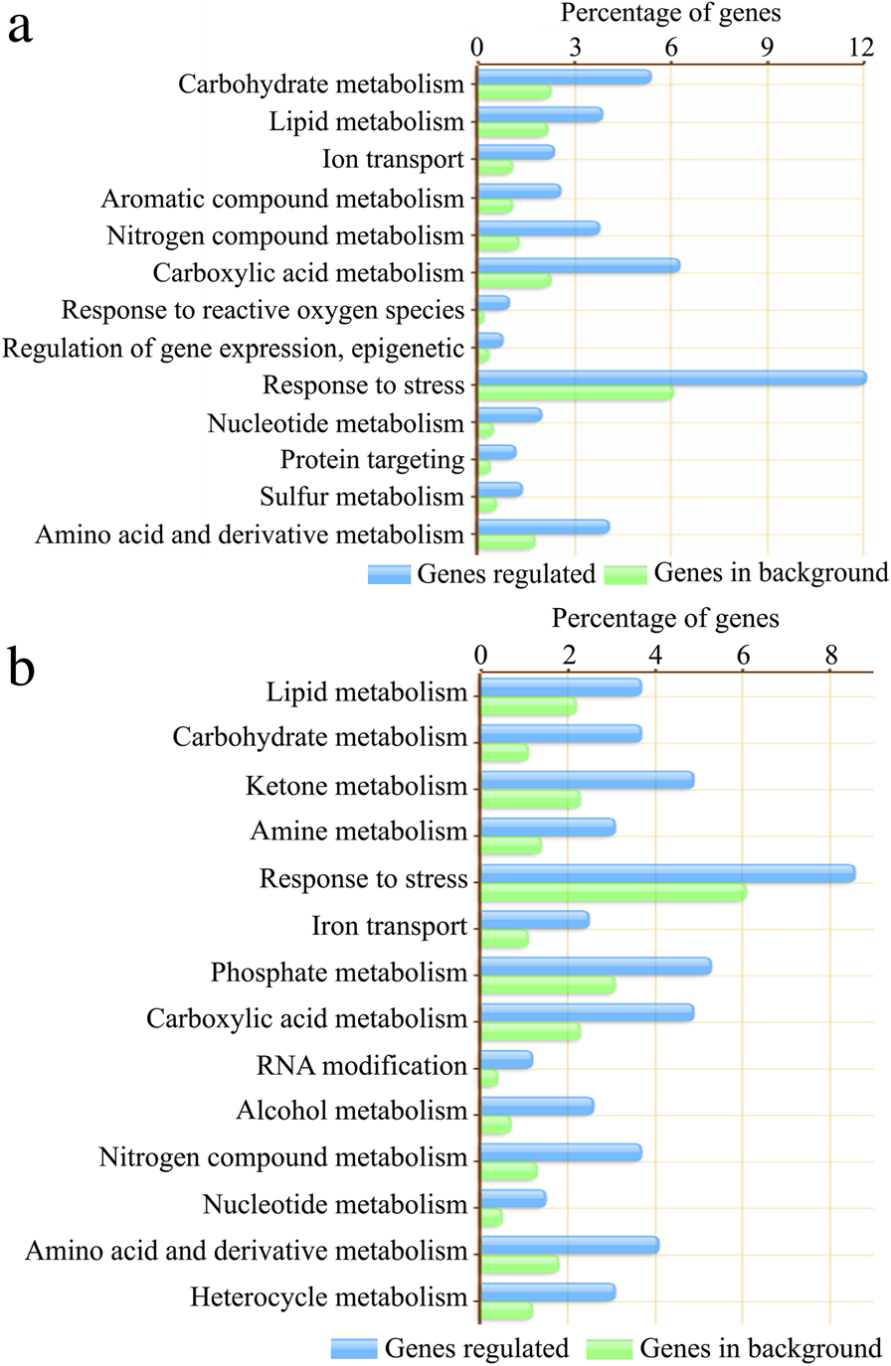
Functional categories of genes responding to alkalinity stress and of those in the full reference set in SR4 and JN177. There are 13 functional categories in SR4 (**a**) and 14 functional categories in JN177 (**b**). Functional categories for gene group that are significantly differentially expressed (shown by *green bars*) and for full reference gene set (*blue bars*) are shown with their percentages

The DGE analysis identified a set of 2,619 and 3028 genes respectively as being transcriptionally altered in SR4 and JN177 through the exposure to alkalinity stress conditions (Additional file 2: Tables S2 and Additional file 3: Table S3). To verify whether the DGE output represented the true variation of the transcripts, twelve genes were randomly chosen for the qRT-PCR amplification. The results were clearly showed that the qRT-PCR data were consistent with the DGE output (Fig. 5). To evaluate the biological functions of alkaline stress responsive genes, GO enrichment analysis were conducted. In all, 13 GO categories were over-represented in SR4 (*P* < 0.01, FDR < 0.05) (Fig. 6a), while 14 GO categories were over-represented in JN177 (*P* < 0.01, FDR < 0.05) (Fig. 6b). The GO categories over-represented in both wheat cultivars were much similar, with most of them for the metabolising different compounds, responsing to stress and iron transport. In SR4 there were also GO categories for response to ROS, epigenetic regulation of gene expression and protein targeting, while RNA modification was over-represented in JN177. In SR4, a group of 62 differential expressed genes were associated with signal transduction and 61 genes in transcription regulation, which included genes encoding calcium sensors, protein kinases, alternative splicing factors and various transcription factors. Another 22 genes were associated with protein modification, among which were proteins involved in phosphorylation, small ubiquitin-related modifier (SUMO) modification, ubiquitin modification and ubiquitin-like modification. There were 37 genes encoding ion transport proteins, 27 genes encoding enzymes participating in lipid metabolism, organic acid metabolism and phenylpropanoid metabolic processes, 23 genes associated with ROS scavenging, 22 genes encoding chaperones, five genes involved in DNA damage repair and five genes involved in chromatin structure remodeling. In JN177, 20 differential expressed genes were associated with RNA modification, including those for pre-mRNA processing factor, RNA helicase and pre-mRNA-splicing factor.

**Fig. 7:**
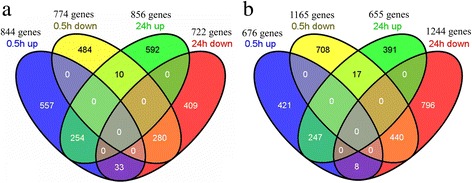
The relationship of differential expressed genes under alkalinity stress conditions treated for 0.5 h and for 24 h. **a** differential expressed genes in SR4, **b** differential expressed genes in JN177. Four ovals represent the four types of differential expressed gene groups which are indicated above the figure. The numbers within one circle denote the number of genes up- or down-regulated by alkali stress condition at one time point. The numbers within multiple circles denote the number of genes up- or down-regulated by alkali stress condition at multiple time points

Exposured the seedlings to alkali stress for 0.5 h, 844 and 676 genes were up-regulated, while 774 and 1165 genes were down-regulated, respectively in SR4 and JN177. Under the 24 h exposure, 856 and 655 genes were up-regulated, while 722 and 1244 genes were down-regulated, respectively in SR4 and JN177 (Fig. 7). Of these differential expressed genes under two conditions, 254 genes were up- and 280 genes down-regulated commonly in both treatments in SR4, while 247 genes were up- and 440 genes down-regulated commonly in both treatments in JN177. A set of 33 and 8 genes were up-regulated in the short exposure treatment, but down-regulated in the long one, while 10 and 17 other genes behaved in the opposite way, respectively in SR4 and JN177. The transcription of a major proportion of the genes was altered either under only the short exposure (557 and 421 up-, 484 and 708 down-regulated respectively in SR4 and JN177), or under only the long exposure (592 and 391 up-, 409 and 796 down-regulated respectively in SR4 and JN177) (Fig. 7). Among the genes affected by the stress at both time point in SR4, ten encoded chaperones and were strongly induced, six encoded heat shock proteins, ten encoded transporter proteins (including four nitrate transporters, a plasma membrane H^+^-ATPase and a vacuolar H^+^-ATPase), nine encoded glutathione transferase and four encoded enzymes involved in phenylpropanoid synthesis.

Most of the genes specifically regulated by the short alkalinity stress treatment in SR4 encoded proteins involved in signal transduction (34 genes), transcription regulation (36 genes), ion transport (14 genes) and molecular chaperoning (12 genes). The 34 signal transduction genes included eight genes encoding calmodulin, calcineurin B-like proteins (CBL), CBL-interacting protein kinase or calcium/calmodulin-dependent protein kinase, five genes encoding mitogen-activated protein kinases, ten genes encoding receptor like kinases, three genes encoding jasmonic acid-related proteins and a homolog of the gibberellin receptor GID1. The 36 regulated transcription regulation genes contained six genes encoding alternative splicing regulators, six genes encoding zinc finger transcription factors, five genes encoding members of the MYB transcription factor family and four genes encoding NAM, ATAF1,2, CUC2 (NAC) transcription factors. The regulated transporter genes were one gene encoding the vacuolar proton-inorganic pyrophosphatase (H^+^-PPase), one gene encoding Ca^2+^/H^+^-exchanging protein and one genes encoding transporters for NO_3_^−^, NH_4_^+^, K^+^, Fe^3+^, Mg^2+^ and Si. Of the 12 chaperones, ten genes encoded small (<40 kDA) heat shock proteins.

The genes regulated by the long exposure time to alkali stress in SR4 were genes encoding proteins associated with the cellular response to nutrition status, ion transport, redox homeostasis balance, histone modification and cellular metabolism. There were 16 genes encoding transporters (four NO_3_^−^ transporters, three aquaporins, two NH_4_^+^ transporters, two SO_4_^2−^ transporters, two Fe-phytosiderophore transporters and one each of K^+^, H_2_PO_4_^−^, Fe^3+^ and Zn^2+^ transporters). In addition, there were 12 genes encoding ROS scavenging enzymes, including SOD, thioredoxin and glutathione transferase. Genes encoding a ROS responsive DNA damage repair-related poly (ADP-ribose) polymerase, an RCD1-like protein [11] and two DNA damage repair proteins were up-regulated, while the set of genes repressed included four genes encoding various histone variants and one gene encoding histone deacetylase 19-like protein. There were 31 genes involved in cellular metabolism were specifically induced, including 12 genes encoding enzymes involved in lipid metabolism, nine genes encoding enzymes in carbohydrate metabolism and five genes encoding enzymes in flavonoid synthesis. Two gibberellin associated genes were up-regulated: one encoded a gibberellin 3-oxidase and the other was a homolog of *SPINDLY*, a negative regulator of GA signalling. Details of all of these regulated genes are listed in Additional file 4: Table S4 and Additional file 5: Table S5.

### The differential response of SR4 and JN177 to alkalinity stress

**Fig. 8:**
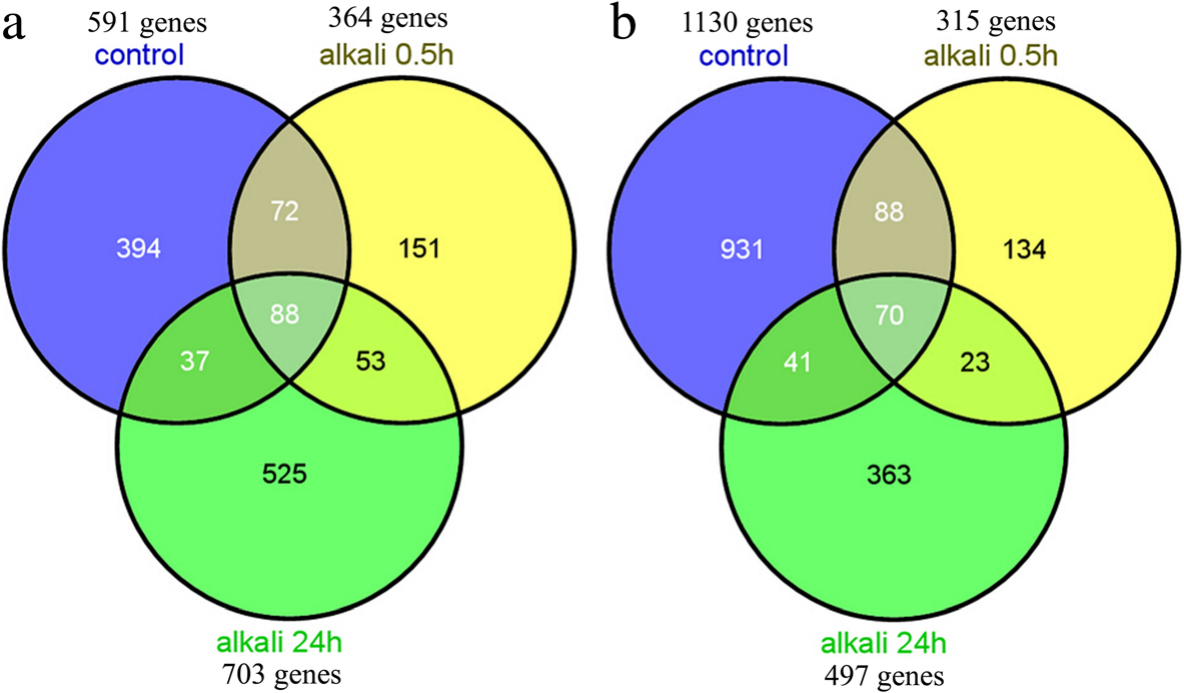
Differential expressed transcripts in SR4 and JN177 under both non- alkalinity and alkalinity stress conditions. **a** Genes transcribed more abundantly in SR4 than in JN177, **b** vice versa

In the absence of alkaline stress, 1,721 genes (591 up- and 1130 down-regulated) showed differentially transcribed in SR4 compared to that in JN177 (Fig. 8). The most well populated GO categories were those involved in transport, transcription regulation, signal transduction, epigenetic modification, cellular metabolism and molecular chaperoning. Among the 31 genes involved in transport, seven genes encoding NO_3_^−^ transporters, six genes encoding aquaporins, two genes encoding NH_4_^+^ transporters, two genes encoding vacuolar H^+^-ATPases, and one gene encoding a plasma memebrane H^+^-ATPase. Among the 28 transcription factor genes, 21 genes were more abundantly transcribed in SR4 than that in JN177. Similarly, nine genes encoding protein kinases were up-regulated in SR4, as well as one gene encoding a jasmonate-induced protein and one gene encoding JAZ protein. There were 17 genes involved in carbohydrate metabolism which were differentially transcribed in SR4 and JN177. The abundance of seven transcripts associated with flavonoid synthesis was less in SR4 than in JN177. All eight differentially transcribed genes involved in epigenetic regulation were down-regulated in SR4; these included three genes encoding histone variants, two genes encoding DNA methyltransferases, one gene encoding histone deacetylase, one gene encoding DNA-(apurinic or apyrimidinic site) lyase and one gene encoding chromatin assembly factor protein.

Following the short alkalinity stress treatment, the abundance of 364 transcripts was higher and that of 315 was lower in SR4 compared to in JN177; the numbers under the long exposure were 703 and 497, respectively (Fig. 8, Additional file 6: Table S6). The majority of genes encoding transporters (for NO_3_^−^, NH_4_^+^, H_2_PO_4_^−^, Fe^3+^ and some other ions) were more highly transcribed in SR4 than in JN177. A set of 13 genes encoding various transcription factors were up-regulated in SR4 in response to the stress. As also was the case in the absence of the stress, the abundance of transcripts was more abundant in SR4 than in JN177 including the genes encoding a plasma membrane H^+^-ATPase, a jasmonate-induced protein and a JAZ protein. Most of the 20 regulated genes encoding ROS scavenging enzymes were more highly transcribed in SR4 than in JN177, including genes encoding SOD, glutathione transferase and dehydroascorbate reductase. The transcription of an *RCD1-like* homolog was also more abundant in SR4 than in JN177. Although the seven genes involved in epigenetic regulation which were differentially transcribed in SR4 than JN177 in the absence of stress were down-regulated in SR4, six of the seven were more strongly induced by the 24 h stress episode in SR4 than JN177.

## Discussion

### SR4 has greater tolerance to alkaline stress than JN177

Root and shoot lengths are both sensitive indices of the plant response to abiotic stress. While the alkalinity stress imposed here was sufficiently strong to compromise the growth of both the wheat seedling shoot and root, SR4 was clearly better able to tolerate with the stress than was JN177 (Fig. 1). The ability of plants challenged with alkalinity stress to take up K^+^ is typically weakened, which also leads to the over- uptaking of the toxic ion Na^+^. The ability of a plant to maintain K^+^/Na^+^ ratio homeostasis has been suggested as a diagnostic of tolerance to both salinity and alkalinity stress. When plants were exposed to alkalinity stress, the low tolerant JN177 plants were less able to maintain their K^+^/Na^+^ ratio than were the SR4 ones. In addition, less MDA was generated in SR4 than in JN177 roots, which implied that a reduced level of plasma membrane damage caused by lipid peroxidation.

### SR4 possesses a high ability in absorbing nutrient ions under alkali stress

The cellular response to alkalinity stress is much less well studied than that to salinity stress. It has been suggested that a high pH environment can inhibit the plant’s capacity to take up NO_3_^−^ and H_2_PO_4_^−^, with consequences for the nutritional status of the plant [19, 20]. The transcriptome analysis revealed that a number of NO_3_^−^, H_2_PO_4_^−^ and SO_4_^2−^ transporters were much more strongly up-regulated in SR4 plants under alkalinity stress condition than in JN177 plants. The qRT-PCR analysis also indicated that a nitrate transporter gene *NRT1* was induced in SR4 under alkalinity stress (Fig. 5c). Moreover, the NO_3_^−^ and H_2_PO_4_^−^ content were much higher in SR4 than in JN177 under alkali stress (Fig. 2k−l). The implication is that the genetic make-up of SR4 allows it, when exposed to alkalinity stress, to better maintain an adequate supply of key anions than can JN177. A high soil pH has a significant impact on the availability of a number of important cations (notably Ca^2+^, Mg^2+^, Zn^2+^ and Fe^3+^), with further negative consequences for the ion homeostasis and nutritional status [19, 21]. Graminaceous plants acquire Fe^3+^ by releasing phytosiderophores able to chelate the ion. Of note is that the constitutive expression in barley of an Fe phytosiderophore transporter gene has a marked ameliorative effect on the plant’s tolerance of alkaline soil [22]. The present analysis revealed that two putative Fe phytosiderophore transporter genes were up-regulated by alkalinity stress in SR4 and that their transcript abundance was higher than in JN177. In addition, a number of other cation transporter genes were induced by alkalinity stress in SR4, and two of these (encoding a Mn^2+^ transporter and a putative Zn^2+^ transporter) were differentially up-regulated in alkalinity stressed SR4. Physiological analysis also indicated that SR4 contained higher levels of Ca^2+^, Mg^2+^, total Fe, Mn^2+^ and NH_4_^+^ than JN177 under alkali stress (Fig. 2c–e, h and i). Taken together, we suggest that SR4 may have a higher ability to absorb both nutrient anions and cations than JN177 under alkaline condition, which may help to explain the better performance of this cultivar upon alkalinity.

### SR4 controls the intracellular and rhizosphere pH levels more effectively than JN177

In addition to its impeding nutrient uptake, a high soil pH can also directly affect the internal pH of the plant [19]. Part of the resistance mechanism against alkalinity stress involves the expenditure of energy to control both the intracellular and rhizosphere pH. Eukaryotic cells have evolved for this purpose various ion pumps, such as plasma membrane proton translocating ATPases (PM H^+^-ATPases), vacuolar H^+^-ATPases (V-ATPases) and proton pyrophosphatase (H^+^-PPases). V-ATPases form part of the cellular machinery responsible for regulating intracellular pH [23–25], while H^+^-PPases act to complement V-ATPase transport activity through the acidification of intracellular compartments [26]. The DGE analysis revealed a number of genes encoding V-ATPase or H^+^-PPase being induced by alkalinity stress; some of these were more abundantly transcribed in SR4 than in JN177. Measurement of intracellular pH also indicated that the root intracellular pH decreased in both alkalinity stressed JN177 and SR4, moreover, it was much lower in SR4 than in JN177 under alkalinity stress (Fig. 3), which coincide with the DGE data. The implication is that SR4 may be better able than JN177, through its use of various V-ATPases and H^+^-PPases, to maintain intracellular pH homeostasis when challenged with alkalinity stress.

The plasma membrane H^+^-ATPases of plant cells establish the pH and membrane potential gradients across the plasma membrane [27], and some recent research has shown that plasma membrane H^+^-ATPase appears to mediate proton secretion during the adaptation of the root to alkaline stress [28]. The protein kinase PKS5 (CIPK11) inhibits the plasma membrane H^+^-ATPase by preventing interaction with 14-3-3 protein, loss-of function *pks5* mutant plants are more tolerant of high external pH due to extrusion of protons to the extracellular space [28]. Moreover, a chaperone J3 (DnaJ homolog 3) activates the plasma membrane H^+^-ATPase by repressing PKS5 [29]. In tomato, the product of *TFT4* (which encodes a 14-3-3 signaling protein) integrates proton efflux and the PKS5-J3 pathway as part of the root’s alkalinity stress response [30]. The present transcriptomic analysis identified the differential up-regulation of one gene in SR4 encoding a plasma membrane H^+^-ATPase gene, both under non-stressed and alkalinity stressed conditions. In addition there were three differentially regulated genes encoding a DnaJ homolog chaperone, as well as one encoding a 14-3-3-like protein. This suggests that the differential transcription may enable SR4 to secrete protons more effectively into the rhizosphere than can JN177, and thereby improve its ability to withstand stress.

### SR4 tissue has a more effective ROS scavenging ability than JN177

Plants have evolved a number of ROS removal mechanisms to avoid excessive cellular damage when high levels of ROS are present [31]. SOD converts O_2_^•−^ radicals to H_2_O_2_ [32], which is in turn decomposed by APX, GPX and CAT [31]. Under alkalinity stress, SR4 showed higher SOD, CAT and APX activity than JN177 (Fig. 4e–g), and the transcript abundance of most of the 20 genes involved in ROS scavenging was also higher in SR4. Direct measurement of ROS showed that the SR4 accumulated less ROS than JN177 (Fig. 4a–c). All these results suggested that SR4 had a powerful ability to prevent the over accumulation of ROS and to protect the cells against ROS caused by alkali stress. It had been concluded that the enhanced salinity tolerance of SR3, the sister line of SR4, also governed by a more active ROS scavenging system than JN177 [6, 9]. It seems like that the higher ROS scavenging ability is the common feature of both SR4 and SR3 in defending salt-alkali stress.

The ROS responsive *RCD1*-like gene *TaSRO1* has been shown to be an important component of the enhanced salinity tolerance of the SR4 sister line SR3, partly due to its ability to regulate ROS homeostasis and partly to maintain DNA integrity [11]. The same gene, along with two others associated with DNA damage repair, was also induced by alkalinity stress, with a stronger level of induction in SR4 than in JN177. It suggested that SR4 might also have stronger ability to maintain DNA integrity under alkali stress, like SR3.

### Involvement of epigenetic modification in the alkalinity stress responses

Since the abiotic stress response involves changes to the transcription of large numbers of genes, global regulation mechanisms are likely to be triggered by the stress. One such mechanism could operate through alterations in the chromatin, involving both non-sequence changes to the DNA (eg through cytosine methylation) and covalent modifications to the histone (eg acetylation, methylation, phosphorylation, ubiquitinylation) or even the replacement of canonical histones by specific variants [33]. Histone variants are known to affect the plant response to both low and high temperature stress, and also are induced by P starvation [34–36]. The incorporation of histone variants depends in part on the action of histone chaperones and chromatin remodeling factors [37, 38]. Some aspects of transcription during episodes of abiotic stress have been shown to be affected by the methylation variants present within specific genes in a range of plant species [39–41]. The somatic hybridization process (as used to generate SR4, see [5]) has been shown to induce wholesale alterations in cytosine methylation patterns, and some of these have been implicated in underlying the heightened salinity tolerance displayed by SR3 [42]. Both cytosine methylation and histone deacetylation variants are believed to be responsible for regulating the level of transcription of certain salinity stress responsive genes in SR3 [42]. The present transcriptomic analysis identified eight genes involved in epigenetic regulation (encoding various histone variants, a DNA methyltransferase, a histone deacetylase and a chromatin assembly factor protein) which were differentially transcribed between SR4 and JN177, and six of these genes were up-regulated by the imposition of alkalinity stress for 24 h and showed higher expression level in SR4 than in JN177. Whether epigenetic modifications are involved in alkali stress responses and its contribution to the alkali tolerance in SR4 will require further investigation.

## Conclusion

Alkali stress was normally closely related to salt stress in soil; however, alkali stress and salt stress are two distinct kinds of stresses for plants. Salt stress consists of the low water potentials and ion toxicities factors, while alkali stress has an added high-pH effect, which is the major feature of alkalinity stress. The high pH surrounding the roots could direct some ions to precipitate, which may destroy the nutrient supply and ion balance around the roots. Moreover, a high soil pH can also directly affect the internal pH of the plant. Bread wheat cultivar SR4 expresses a superior tolerance to alkaline stress than its parent cultivar JN177. This study showed that this superiority are due to its capability in taking up nutritionally important anions and cations, in having a more active ROS scavenging ability and also probably in regulating both the plant’s internal pH and that of its rhizosphere. Since higher ROS scavenging ability was also involved in the salinity tolerance of another somatic introgression wheat cultivar SR3, considering the extra high pH effect of alkalinity stress compared with salinity stress, the high absorbing ability for nutrient ions and the good regulating ability for intracellular and rhizosphere pH might be more important for alkali tolerance of wheat. All of the evidences provide clues for uncovering the tolerance of SR4 to the alkalinity stress and broaden our understanding of the mechanism of wheat to alkalinity.

## Methods

### Plant materials and growth conditions

The bread wheat (*Triticum aestivum*) cultivar Shanrong No. 4 (SR4) used in this study is a derivative of an asymmetric somatic hybrid between bread wheat cultivar JN177 and tall wheatgrass (*Th. ponticum*) created by Dr. Guangmin Xia, and the detail information about asymmetric somatic hybridization was described in Xia’s report [4]. JN177 and SR4 grains were germinated on moist filter paper for two days at 23°C and then transferred to half-strength Hoagland’s liquid medium under a 16 h photoperiod, 50% relative humidity and 300 μmol m^−2^s^−1^ photon flux density light. Alkalinity stress was applied after nine days by adding 100 mM mixed salts (NaHCO_3_:Na_2_CO_3_ in a molar ration at 9:1, pH 8.90). The solution was changed daily to avoid anoxia. After ten days of exposure to the alkalinity stress, measurements were made for shoot length, root length and seedling fresh and dry weight from 20 seedlings of each cultivar, with triple replicates. Image J software (National Institutes of Health) was used to measure the length of the roots of the two wheat cultivars under non-alkaline and alkaline stress conditions. At the three leaf stage (21 d after germination), the hydroponic solution was supplemented with 100 mM mixed salts (NaHCO_3_:Na_2_CO_3_ in a molar ration at 9:1, pH 8.90). After 24 h exposure to alkaline stress, the roots of seedlings in the alkalinity treatment and control were harvested for detecting the contents of MDA, O_2_^•−^, H_2_O_2_, ions and the activities of APX, GPX, POD, SOD and CAT. The experiment was replicated three times. Seedlings used as a source of RNA extraction were grown to the three leaf stage in half strength Hoagland’s liquid medium. After either a 0.5 h or a 24 h exposure to 100 mM of the mixed alkali salts (NaHCO_3_:Na_2_CO_3_ by 9:1 molar ratio, pH 8.90), the roots of both treated and non-treated seedlings were snap-frozen in liquid nitrogen and stored at −80°C until required. Each treatment was replicated three times.

### Physiological indicators of stress

The root MDA contents were assessed following Liu et al. [9]. The root ion contents were determined following Yang et al. [19]. POD activity was measured following Ranieri et al. [43]. The root H_2_O_2_ content and activities of SOD, GPX and CAT were assayed using commercial kits provided by Beyotime (Beyotime, Shanghai, China). The activity of APX was assayed using commercial kit provided by Solarbio (Solarbio, Beijing, China). To monitor the accumulation of O_2_^•−^ radicals, roots were infiltrated with 10 mL nitro blue tetrazolium (NBT) solution (0.5 mg mL^−1^ NBT supplied in 10 mM potassium phosphate buffer, pH 7.8) for 1 h in the dark, then staining reaction was stopped by adding an excess of 70% (v/v) methanol. The roots were rinsed in fresh 70% (v/v) methanol and then observed and photographed under a stereomicroscope. The strength of the NBT signal was estimated from a digital image of the stained tissue with ImageJ software (National Institutes of Health). The polymerization product after NBT staining can reflect the O_2_^•−^ content, thus the relative superoxide content of each sample can be represented by the average pixel density in the root area of the photograph.

### Intracellular pH

The intracellular pH was monitored with BCECF. The roots of JN177 and SR4 were incubated for 30 min in the dark at 22–24°C in incubation buffer (pH 6.2) containing 10 mM MOPS, 50 mM KCl, 1 mM MgCl_2_, 0.05% pluronic F-127 and 10 μM BCECF-AM. Loading was terminated by rinsing the roots three times in incubation buffer lacking the dye. BCECF-AM loaded into the cytoplasm was hydrolyzed to BCECF by esterase. The intracellular pH was monitored by measuring the fluorescence of BCECF with a laser scan confocal microscope (LSM700, Zesis, Germany). The excitation wavelength was 488 nm and emission wavelength was 525 nm. The pH showed a linear relationship with fluorescence intensity over the pH ranges 6.4-7.6. For pH calibration, the wheat roots were incubated in the dark in calibration buffer (pH6.6, 7.0, 7.2, 7.4 and 7.6) containing 10 mM MOPS, 130 mM KCl, 1 mM MgCl_2_, 10 mM NaCl, 0.05% pluronic F-127, 10 μg/mL nigericin and 10 μM BCECF-AM.

### RNA sequencing

Tag libraries were constructed from root mRNAs extracted from alkalinity-stressed (both 0.5 h and 24 h exposure times) JN177 and SR4 plants, as well as from the non-stressed roots of both cultivars. Magnetic beads coated with polyT were used to isolate the mRNA fraction of a 30 μg aliquot of total RNA. Double strand cDNA synthesized from this mRNA was digested with *Nla*III, an enzyme which cleaves at the sequence CATG. *Nla*III-restricted 3′ cDNA fragments were purified by Oligo(dT) beads, then the Illumina adaptor 1 is ligated to the sticky 5′ end, which retains the CATG site. The fragments were further digested with *Mme*I (this enzyme cleaves 17 bp downstream of the CATG site), then ligated to Illumina adaptor 2 to construct a 21 bp tag library with different adaptors at both ends. After linear PCR amplification based on the primer pair corresponding to the adaptors, the amplified samples were purified by polyacrylamide gel electrophoresis. The quality of the constructed library was analyzed by Agilent 2100 Bioanaylzer and ABI StepOnePlus Real-Time PCR System. The tag library was finally sequenced using a HiSeq 2000 device (Illumina). The short-read sequence data from the six libraries are respectively deposited in NCBI Sequence Read Archive (SRA, http://www.ncbi.nlm.nih.gov/sra) with accession number SRR3223630, SRR3223636, SRR3223646, SRR3223672, SRR3223674 and SRR3223675. Adaptor tags (the raw sequences containing 3′ adaptor sequences), empty reads (reads with only 3′ adaptor sequences but no Tags), low-quality tags (tags with ambiguous base calls), tags of unusual length (longer or shorter than 21 nt) and nonredundant tags (tags with a copy number less than 2) were removed from the raw data, yielding a dataset consisting of clean tags. Subsequently, the clean tags were classified according to their copy number in the library, their percentage of total clean tags was calculated, and the saturation of the library was analyzed.

### Differential transcription

The frequency of each tag in the various cDNA libraries was used to infer differential transcription. To identify genes involved, the reference sequences from NCBI wheat EST collection (http://ftp.ncbi.nih.gov/repository/UniGene/Triticum_aestivum/Ta.seq.uniq.gz) or IWGSC wheat genomic DNA sequences (ftp://ftpmips.helmholtz-muenchen.de/plants/wheat/IWGSC/genePrediction_v2.2/) were searched for CATG sites, all the possible CATG + 17 nt sequences were used as a reference tag library for following clean tags mapping. All clean tags were mapped to the reference tag library with perfect match or allowing a 1 nt mismatch (there might be SNPs between different varieties) [44], conducting by a Perl program through string matching. Tags identifying more than one gene were removed, and the number of remaining unambiguous tags was normalized to TPM (number of transcripts per million clean tags). To identify differentially expressed genes (DEGs), an algorithm to identify differential transcript accumulation between different samples (alkalinity vs no alkalinity treatment, SR4 vs JN177) was used according to Audic and Claverie [45]. A false discovery rate (FDR) threshold of 0.001 and a log2 ratio threshold of 1 were applied as the criteria for declaring differential transcription [46]. Gene ontology (GO) assignments and enrichment analysis was conducted to assign biological process, cellular component and molecular function through hypergeometric test to map all differentially expressed genes to terms in GO database (http://amigo.geneontology.org/amigo), looking for significantly enriched GO terms in DEGs comparing to the genome background.

#### Quantitative real-time PCR (qRT-PCR)

Total root RNA extracted using the TRIzol reagent (Invitrogen) provided the template for a qRT-PCR assay. The cDNA was synthesized from 2 μg DNase-treated total RNA based on M-MLV reverse transcriptase (TaKaRa) in accordance with the manufacturer’s protocol. Each 10 μL qRT-PCR contained 5 μL SYBR Green, 5 μM of each primer and 1 μL template. The reference sequence was a segment of the wheat *Actin* gene (AB181991). Relative expression level was calculated using the 2^-ΔΔCt^ formula. For each of the three biological replicates, three technical replicates were included. All relevant primer sequences are listed in Additional file 7: Table S7.

## Additional files

Additional file 1: Table S1. Statistics analysis of the six DGE tag libraries constructed from the seedling roots of JN177 and SR4 mapped to wheat genomic DNA sequences. (DOC 32 kb)
Additional file 2: Table S2. Differentially expressed genes in SR4 after alkalinity stress. Total number of genes differentially up- and down-regulated in roots under 100 mM alkaline stress treatment compared with the sample without alkalinity stress (*P* < 0.05, *q* < 0.15). (XLS 1142 kb)
Additional file 3: Table S3. Differentially expressed genes in JN177 after alkalinity stress. Total number of genes differentially up- and down-regulated in roots under 100 mM alkaline stress treatment compared with the sample without alkalinity stress (*P* < 0.05, *q* < 0.15). (XLS 1340 kb)
Additional file 4: Table S4. Differentially expressed genes at different time point of alkalinity treatment in SR4. (XLS 4177 kb)
Additional file 5: Table S5. Differentially expressed genes at different time point of alkalinity treatment in JN177. (XLS 1417 kb)
Additional file 6: Table S6. Differentially expressed genes between SR4 and JN177 under non-alkalinity or alkalinity stress. (XLS 1230 kb)
Additional file 7: Table S7. The primer sequences for qRT-PCR analysis. (XLS 40 kb)

## Abbreviations

ABA: Abscisic acid
APX: Ascorbate peroxidase
CAT: Catalase
CBL: Calcineurin B-like proteins
CDPK: Calcium/calmodulin-dependent protein kinase
CIPK: CBL-interacting protein kinase
DGE: Digital gene expression
DHAR: Dehydroascorbate reductase
GA: Gibberellic acid
GO: Gene ontology
GPX: Glutathione peroxidase
HSP: Heat shock protein
JAZ: Jasmonate ZIM domain-containing protein
MAPK: Mitogen-activated protein kinase
MDA: Malondialdehyde
MYB: Myeloblastosis
NAC: NAM, ATAF1,2, CUC2
NBT: Nitro blue tetrazolium
POD: Peroxidase
qRT-PCR: Quantitative real-time PCR
RLK: Receptor like kinase
ROS: Reactive oxygen species
SOD: Superoxide dismutase
SUMO: Small ubiquitin-related modifier
