# Supplementary material for: Transcriptome profiling reveals the genetic basis of alkalinity tolerance in wheat
Source: BMC Genomics. 2017 Jan 5;18:24. doi: 10.1186/s12864-016-3421-8 (PMC5217398; doi:10.1186/s12864-016-3421-8)
Supplement: Additional file 1: Table S1. — Statistics analysis of the six DGE tag libraries constructed from the seedling roots of JN177 and SR4 mapped to wheat genomic DNA sequences. (DOC 32 kb) [file 12864_2016_3421_MOESM1_ESM.doc]

**Table S1. Statistics of the six DGE tag libraries constructed from the seedling roots of JN177 and SR4 mapping to wheat genome sequences.**

| Mapping to IWGSC database | JNCK | JN0.5 | JN24 | SRCK | SR0.5 | SR24 |
| --- | --- | --- | --- | --- | --- | --- |
| Total number of clean tags | 5943058 | 5712643 | 5487894 | 5692072 | 5633349 | 5525666 |
| Clean tags mapping to gene | 4264438 | 3994320 | 3713992 | 3921164 | 3981843 | 3887805 |
| % of clean tags mapping to gene | 71.75% | 69.92% | 67.68% | 68.89% | 70.68% | 70.36% |
| Unambiguous tags mapping to gene | 1267841 | 1284077 | 1158247 | 1233959 | 1300910 | 1177607 |
| % of unambiguous tags mapping to gene | 21.33% | 22.48% | 21.11% | 21.68% | 23.09% | 21.31% |
